# The smallest quaternary ammonium salts with ether groups for high-performance electrochemical double layer capacitors†
†Electronic supplementary information (ESI) available. See DOI: 10.1039/c5sc02755a



**DOI:** 10.1039/c5sc02755a

**Published:** 2015-11-30

**Authors:** Taihee Han, Min-Sik Park, Jeonghun Kim, Jung Ho Kim, Ketack Kim

**Affiliations:** a Department of Chemistry , Sangmyung University , Seoul 110-743 , Republic of Korea . Email: Ketack.kim@smu.ac.kr; b Advanced Batteries Research Center , Korea Electronics Technology Institute , Seongnam 463-816 , Republic of Korea; c Institute for Superconducting and Electronic Materials , Australian Institute for Innovative Materials , University of Wollongong , North Wollongong , NSW 2500 , Australia

## Abstract

Electrochemical double layer capacitors (EDLCs) are energy storage devices that have been used for a wide range of electronic applications. In particular, the electrolyte is one of the important components, directly related to the capacitance and stability. Herein, we first report a series of the smallest quaternary ammonium salts (QASs), with ether groups on tails and tetrafluoroborate (BF_4_) as an anion, for use in EDLCs. To find the optimal structure, various QASs with different sized head groups and ether-containing tail groups were systematically compared. Comparing two nearly identical structures with and without ether groups, QASs with oxygen atoms showed improved capacitance, proving that ions with oxygen atoms move more easily than their counterparts at lower electric fields. Moreover, the ether containing QASs showed low activation energy values of conductivities, leading to smaller *IR* drops during the charge and discharge processes, resulting in an overall higher capacitance.

## Abstract 2

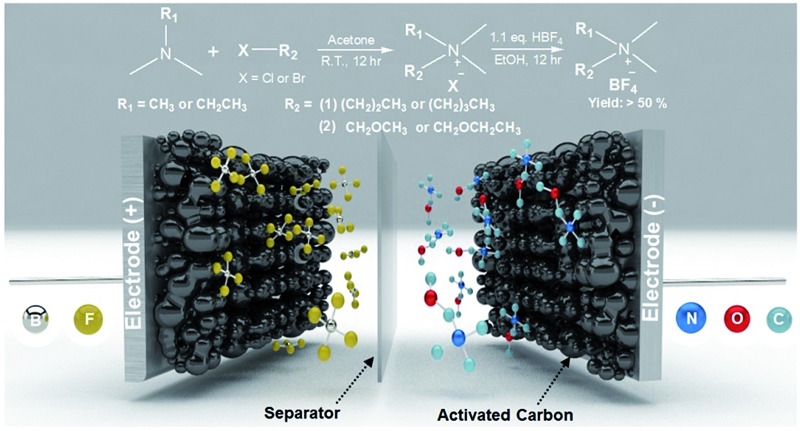
The smallest quaternary ammonium salts (QASs) with ether groups on tails and tetrafluoroborate (BF_4_) as an anion exhibit high performance in electrochemical double layer capacitors (EDLCs).

## Introduction

Electrolytes have been regarded as one of the most important components for high performance electrochemical double layer capacitors (EDLCs). Among the various electrolytes, organic electrolytes have the advantages of a high operation potential window and the use of low cost materials such as Al electrodes in devices.^2^ These can improve not only the energy and power densities but also the cost-effectiveness of production. Quaternary ammonium salts (QASs) are superior to any other salts in nonaqueous solutions due to their facile synthesis, inexpensive production, and good electrochemical stability up to 3 V.^3,4^ Among QASs, tetraethylammonium tetrafluoroborate (TEA BF_4_) is the most common salt that meets the requirements for cost-effective performance toward practical application. By controlling the pore size of carbon materials, the capacitance of EDLCs can be maximized.^5–7^ However, implementing fine control of the pore size in materials such as carbide-derived carbons,^8,9^ nanotubes,^10,11^ and metal-organic frameworks,^12–17^ in commercially available activated carbon to obtain very narrow pore size distributions would be difficult and lead to additional production costs. Alternatively, utilizing smaller ions^18,19^ and cyclic ions^20,21^ to further improve the capacitance of EDLCs is suggested as an effective approach, because such ions can travel through a wide range of pore sizes in activated carbons during adsorption and desorption. Additionally, ions that are smaller than those of TEA BF_4_ can reach deeper and narrower pores than TEA BF_4_ and thus increase the usable surface of activated carbons, and thereby, high-performance capacitance. In addition to the size control of ions, adding heteroatoms or functional groups to the carbon chains of the quaternary ammonium ions dramatically changes their physicochemical properties.^22^ Unfortunately, most functional groups and heteroatoms added to these ions are electrochemically unstable. Although a smaller cation size will not necessarily lead to improved capacitance, increasing the cation size does induce undesirable capacitance loss. This is because large cations are more difficult to transfer due to their size and high viscosity.^23,24^ By replacing a methylene (CH_2_) moiety with an oxygen atom in the middle of the alkyl skeleton, an ether group with a quite similar size compared to a methyl group can be inserted. Furthermore, ether groups in molecules are relatively stable against electrochemical reactions. It is well-known that poly(ethylene glycol) is widely used as an element of gel electrolytes,^25^ and carbonates that contain ether groups are used as the main solvents in Li-ion batteries.^22,26^ Rennie *et al.* reported that ether group-containing phosphonium bis(trifluoro-methanesulfonyl)imide ionic liquids show improved performance in EDLCs by introducing ether branches on the salts, affecting molecular flexibility and a denser packing of ions due to a small electronegative region in the cation structure.^27^

Several ether group-bearing QASs show good thermal stability of EDLC operation in propylene carbonate. The dependence of anions on the cell properties has been observed.^28^ In addition, ether branches on the cation decrease the viscosity of ionic liquids,^27,29^ which is another advantage of introducing ether groups on cations. Interestingly, when an ether group exists on an ammonium ion, the properties of the ion can be strongly influenced by the oxygen atom. The oxygen atom, which has two electron pairs, can change the ionic characteristics of the ions on carbon electrodes compared with the characteristics observed when CH_2_ is present on the alkyl chains. If there is a strong attraction to, or repulsion away from the electrodes, these behaviors can be observed in the curves of capacitance *vs.* time of EDLCs during the charge and discharge processes. The aim of this work is to find a suitable QAS in both size and chemical moiety for high performance EDLCs and to understand the electrochemical behaviors of ions containing oxygen atoms on ammonium salts used for EDLCs by discussing their physical properties and adsorption/desorption kinetics. In this study, therefore, we prepared pairs of oxygen- and methylene-bearing QASs with BF_4_^–^ at high yield (above 50% after recrystallization), in which the size effect on the EDLC performance is negligible.

## Results and discussion

**Fig. 1:**
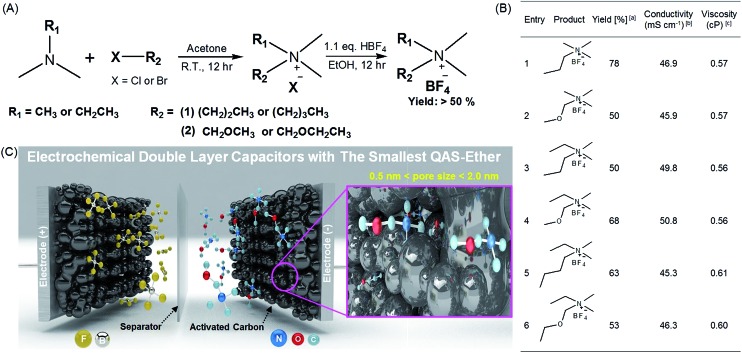
Synthesis of the quaternary ammonium salts (QASs) and their application to electrochemical double layer capacitors (EDLC) with high stability and performance. (A) Synthetic route for various QASs possessing alkyl and ether groups. (B) The summary of synthesized QASs and their physicochemical properties. ^a^Calculated after recrystallization. ^b^1.0 M solutions of QASs in acetonitrile (AN). ^c^1.0 M solution of QASs in AN. (C) Schematic illustration of high-performance EDLC devices using entry 2 with ether groups as the smallest QAS and highly porous activated carbon (MSP20) with a surface area of 2236.3 m^2^ g^–1^ (see Fig. S2 and Table S1†).^1^

**Fig. 2:**
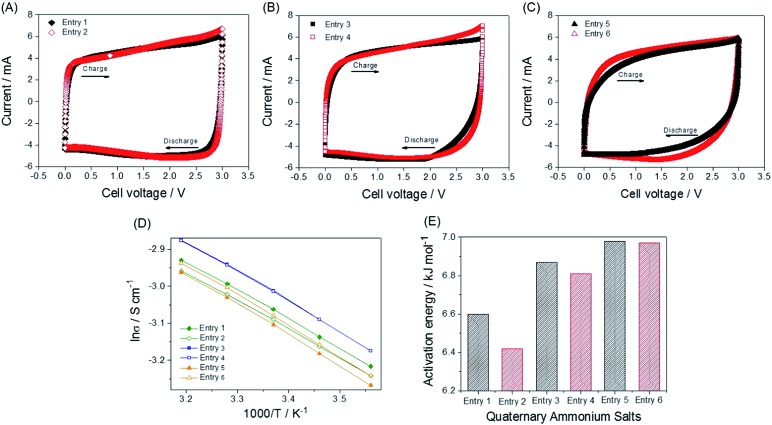
CVs and calculations of the activation energy for all the electrolytes. CVs of (A) entry 1 and entry 2, (B) entry 3 and entry 4, and (C) entry 5 and entry 6 at a scan rate of 5 mV s^–1^. Coin cells (two-electrode cells) are used to obtain the voltammograms. (D) Logarithmic Arrhenius plot of conductivity values. The values are obtained from conductivity measurements in the range of 8 to 40 °C. (E) Activation energy values for the ionic conductivities of the electrolytes in 1.0 M electrolytes.

**Fig. 3:**
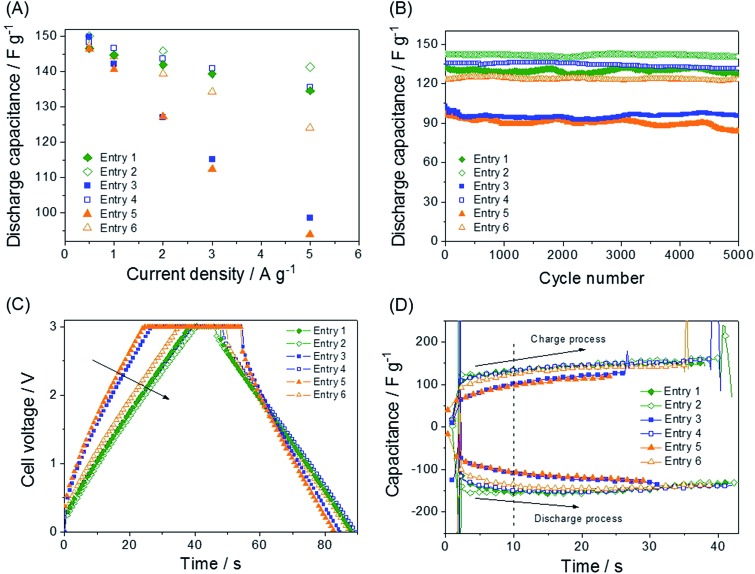
Electrochemical characterizations of the QASs. (A) Galvanostatic capacitance *vs.* current density for the comparison between the ether salts and alkane salts. The capacitance values are the average of three measurements and were obtained from the slope of the range 0–3.0 V. (B) Cycle life tests with the electrolytes. Current density is 5 A g^–1^. Galvanostatic charge and discharge curves. The current density is 5 A g^–1^. (C) Potential *vs.* time and (D) capacitance *vs.* time.

The synthetic routes and structures of the salts and their EDLC device structure are shown in Fig. 1. Six BF_4_ QASs are paired to create three groups of structures differing only by the presence or absence of an oxygen atom in the alkane chains of the cations. All the cyclic voltammograms (CVs) shown in Fig. 2A–C exhibit only capacitive currents, which are the only elements reflected in the capacitance values. Cycle life tests with these electrolytes prove that the electrolysis of the electrolytes was not severe within the given potential range (Fig. 3B). The CVs of the electrolytes containing entry 1 and entry 2 are compared in Fig. 2A. We found that the CV of the ether salt (entry 2) is more rectangular than that of the alkane salt (entry 1). A rapid current increase in the ether salt was evident at the beginning of both the charge and discharge processes, indicating that the ions of the ether salt move faster than those of the alkane salt. For the other pairs described in Fig. 2B and C, similar electrochemical behaviors were observed. The ether salts exhibited a sharper current increase than the alkane salts at the beginning of both the charge and discharge processes. However, the rectangular shape of the CVs becomes increasingly distorted as the cation size increases. This is because smaller ions can move faster than larger ions in the micropores of the activated carbon.^18^ The rapid response of the ions in the ether salts implies that the oxygen atoms on these salts facilitate adsorption and desorption more rapidly than the alkane salts. If the strengths of adsorption and desorption on the carbon electrode are not balanced, the rectangular shape of either the charge or the discharge will be distorted. Thus, this finding reveals that both the charge and discharge rates of ether salts are rapid and well balanced. Fig. 3A shows the galvanostatic capacitance of the electrolytes containing ether salts and alkane salts at different current densities. According to the comparison, the ions of the ether salts retained a higher capacitance than those of the corresponding alkane salts. This difference can be reasonably explained by the fact that agile ions are more likely to maintain the capacitance as the current density is increased. The electrolytes of the ether salts showed a much higher discharge capacitance than those of the alkane salts, even at a high current density of 5 A g^–1^. Among the electrolytes, the smaller ions showed relatively rapid responses to the current variation, maintaining higher capacitance. This is because the smaller ions can move into the narrow pores more easily. Note that the agile small ions can store relatively high levels of energy in micropore-based carbon electrodes. In practice, the large alkane ions, entry 3 and entry 5, exhibit the lowest capacitance values among the electrolytes investigated in our work. To examine the detailed movement of the ions, the potential *vs.* time curves for each electrolyte during the galvanostatic charge/discharge processes are compared in Fig. 3C. Because the common anion, BF_4_^–^, is much smaller than any other cation in the electrolytes,^30^ it can move within the pores more easily than the cations. Therefore, the charge and discharge processes are primarily dependent on the properties and structures of the cations rather than those of the anions. The cell capacitance (eqn (1) in Farad) was calculated from the current, *I* (in ampere (A)), and the slope of the discharge curve (Δ*V*/Δ*t*). Electrolytes entry 3 and entry 5 showed the lowest capacitance and the shortest charging time of all the electrolytes (Fig. 3A, C and D).

1 *C* = *I*/(Δ*V*/Δ*t*)

The charge duration at a current density of 5 A g^–1^ followed the order entry 2 > entry 1 ≈ entry 4 > entry 6 > entry 3 > entry 5. This order fully agrees with that of the capacitance shown in Fig. 3A.

Additionally, the electrolytes with low capacitance values exhibit larger *IR* drops during both the charge and discharge processes because of the higher resistance in initiating the charge and discharge processes. In this respect, more effort is required for the electrolytes entry 5, entry 3, and entry 6 to enter and exit the pores than entry 1, entry 4, and entry 2. The values of the *IR* drop for each process are listed in Table 1, obtained from Fig. 3C. The enlarged curves of Fig. 3C are shown in Fig. S1† so that the *IR* drops for individual curves can be observed more clearly. The capacitance contribution during charge or discharge can be obtained from the differential curve of Fig. 3C, [*I*/(d*V*/d*t*)] *vs.* time (*t*), which is the plot of capacitance (*C*) *vs.* time. The curve of the specific capacitance *vs.* time (Fig. 3D) is obtained. The specific capacitances *C*_am_ in Farad per gram of electrode (F g^–1^) for all samples were obtained from eqn (2).

2 *C*_am_ = 2*C*/*m*_am_

where *m*_am_ is the mass of active material (AC) in an electrode. The durations of the charge processes are shorter than those of the counter discharge processes, because the constant voltage area (3.0 V) is not included in the derivative. The electrolytes that are able to move rapidly (entry 2, entry 4, and entry 1) showed much higher capacitance than entry 5, entry 3, and entry 6 at the beginning of both the charge and discharge processes. In both the charge and discharge processes in which the influence of ionic movement resistance (*IR* drop) is strong, within 10 s, the capacitance values of cells containing entry 5, entry 3, and entry 6 electrolytes were observed to fall behind those of the cells containing entry 2, entry 4, and entry 1. Electrolyte entry 2 responds rapidly to reach its maximum capacitance, and the agility of the entry 2, entry 4, and entry 1 ions is indicated as relatively small *IR* drops. Because the sizes of the compared pairs (*e.g.*, entry 1 and 2, entry 3 and 4, and entry 5 and 6) are almost the same, the size effect on the ionic movement should not be significant. For further inspection of the ionic movement in the electric field, the ionic conductivity and viscosity values of the electrolytes were measured.

**Table 1:** *IR* drop values of charge and discharge processes

Process	Entry 1	Entry 2	Entry 3	Entry 4	Entry 5	Entry 6
Charge (V)	0.219	0.178	0.364	0.217	0.378	0.280
Discharge (V)	0.182	0.134	0.292	0.168	0.300	0.222

The ionic conductivity and viscosity values are not proportional to the cation size. As the ion size increases, the ionic conductivity also increases because the solvated radius decreases. Conversely, as the ion size increases, the viscosity also increases because of the longer alkyl chains. Because of the combined effect of solvated ion size and viscosity, the mid-sized ions exhibit the highest conductivity, as can be seen in Fig. 1B. The largest solvated ions, entry 1 and 2, and the longest alkyl chain ions, entry 5 and 6, showed lower conductivities than entry 3 and 4. Based on the results shown in Fig. 1B, ions with the same size are paired to create groups with similar conductivities and viscosities. However, the physical properties in Fig. 1B do not correspond to the capacitance values in Fig. 3A and are not proportional to the ion size. In addition, the bulk solution conductivities of the electrolytes barely influence the ionic transfer behavior in the micropores of the activated carbon because of the de-solvation of the ions.^5,8,31,32^ The relationship between conductivity/viscosity and the resulting capacitance may vary depending on the degree of de-solvation, which is governed by the pore size of the activated carbon.^33^ To explain the rapid response of ions in the early electric field shown in Fig. 3C and D, it is appropriate to discuss the activation energy values of the conductivities.^34^ Ions that overcome the activation energy at a low potential level (during the early stages of charge and discharge) respond rapidly to the electric field. The activation energy values were obtained from the logarithmic Arrhenius plot of the conductivity values *vs.* temperature in Fig. 2D and the Arrhenius equation (eqn (3)).

3 *σ* = *σ*_0_e^–*E*_a_/*RT*^

The calculated activation energy values are listed in Table 2 and Fig. 2E. The smaller ions exhibit lower activation energies than the larger ions. All the ether ions have lower activation energy values than the corresponding alkane ions. The difference in activation energy values between the ether electrolytes and the alkane electrolytes decreases as the ionic size increases. As the ion size increases, the activation energy for ionic transport also increases, which results in sluggish ionic movement. Because ions with lower activation energy values migrate to and from the electrode more easily during the early stages of the charge and discharge processes (Fig. 3D), the activation energy influences the capacitance at lower electric fields. As the charge and discharge processes proceed, the increased electric field allows all ions to overcome the activation energy. Indeed, ions that respond rapidly at all levels of the electric field result in overall higher capacitance values. The activation energy values explain why some ions in groups of ions with the same size migrate more easily and why the ether ions provide higher capacitance than their corresponding alkane ions. As the comparison of ions in different groups shows, for example, entry 1 and 4, the activation energy is not useful for understanding the order of capacitance values. The smaller ions in the same series (ions in alkane salts or ions in ether salts) are relatively rapidly transported to and from the electrode because of their low activation energy values, as observed in Fig. 3A, C and D. Additionally, the small sizes of these ions lead to better capacitances than larger ions because small ions can penetrate into narrow spaces that large ions cannot reach. The low activation energy of the conductivity provides conditions that allow ions to respond rapidly to the increasing electric field, which results in fast ionic adsorption and desorption during the charge and discharge processes (low *IR* drop). The electrolyte with entry 1, the smallest alkane salt in the work, was reported to have a higher capacitance than those with TEA BF_4_ and triethylmethylammonium BF_4_ (TEMA BF_4_).^18^ Therefore, at least three QASs in this work, entry 1, 2 and 4, can potentially provide higher capacitance than TEA BF_4_ and TEMA BF_4_ according to the capacitance values in Fig. 3A. Two ether salts, entry 2 and 4, showed superior capacitance to the most common QASs, such as TEA BF_4_ and TEMA BF_4_. An accelerated durability test was performed to evaluate whether the ether salt is stable enough to be used in commercial cells at 3.0 V operation. Results of floating durability tests, such as values of capacity retention and internal resistance changes are listed in Table S2.† Even though cycle test results at ambient temperature in Fig. 3B are very stable up to 5000 cycles, the floating test reveals the unstable behavior (lower capacity retention and higher resistance increase) of entry 2 compared to TEA BF_4_ and entry 1. Under severe test conditions, the ether salts can be less stable than the alkane salts. Modification of functional groups on the electrode surface and strict removal of H_2_O in the electrode and electrolytes are further research efforts to come closer to the practical application of the ether salts in EDLC.

**Table 2:** Activation energy values of the ionic conductivities in 1.0 M electrolytes

Electrolyte	Entry 1	Entry 2	Entry 3	Entry 4	Entry 5	Entry 6
*E*_a_ (kJ mol^–1^)	6.60	6.42	6.87	6.81	6.98	6.97

## Conclusions

Although EDLCs have been recognized as durable and inexpensive energy storage devices, their specific capacitance and discharge power must be further improved to meet the requirements of energy storage applications such as portable electronic devices and hybrid and electric vehicles. By substituting the conventional TEA BF_4_ with smaller cations, the capacitance of EDLCs can be remarkably improved. Furthermore, ether group-substituted cations clearly exhibit an improved capacitance compared with their corresponding alkane-substituted cations. The small ether-substituted cations exhibit better capacitance values than other cations. The value of the activation energy for conductivity explains why small cations and ether-substituted cations are more easily transported in the electric field than large cations and normal alkane cations. This new series of electrolytic salts can be applied to produce high-energy and high-power EDLCs when utilized alone or as mixed electrolytes for activated carbon with a high surface area and possessing small pores.
