# Supplementary material for: The smallest quaternary ammonium salts with ether groups for high-performance electrochemical double layer capacitors
Source: Chem Sci. 2015 Nov 30;7(3):1791–6. doi: 10.1039/c5sc02755a (PMC5604401; doi:10.1039/c5sc02755a)
Supplement: Supplementary file 1 [file SC-007-C5SC02755A-s001.pdf]

Electronic Supplementary Material (ESI) for Chemical Science.
This journal is © The Royal Society of Chemistry 2015

Electronic Supplementary Information

The Smallest Quaternary Ammonium Salts with Ether Group for High-performance Electrochemical Double Layer Capacitors

Taihee Han,[a] Min-Sik Park,[b] Jeonghun Kim,[c] Jung Ho Kim,[c] Ketack Kim*[a]

[a] Department of Chemistry, Sangmyung University, Seoul 110-743, Republic of Korea

[b] Advanced Batteries Research Center, Korea Electronics Technology Institute, Seongnam 463-816, Republic of Korea

[c] Institute for Superconducting and Electronic materials, Australian Institute for Innovative Materials, University of Wollongong, North Wollongong, NSW 2500, Australia

Experimental Section

Chemicals: N,N,N-Trimethyl-N-methoxymethylammonium tetrafluoroborate (Entry 2) was synthesized by the following procedure. Trimethylamine (13% in tetrahydrofurane, Tokyo Chemical Industry Co. Ltd.) and chloromethylmethylether (Tokyo Chemical Industry Co. Ltd., 95%) was mixed and stirred for 12 h in a 1:1.1 mol ratio in acetone (Daejung Chemicals & Metals Co.) at ambient temperature. At the beginning of the reaction, the use of an ice bath for 1 h is necessary because the reaction is exothermic. The product, Entry 2, was collected by filtration. The product and HBF_4 (50% in H_2O, Alfa Aesar) were reacted in a 1:1.1 mol ratio in ethanol for 12 h. Solvent and unreacted components were removed under vacuum. Brown liquid was obtained, from which Entry 2 was precipitated with n-butanol (Daejung Chemicals & Metals Co.). The final product was recrystallized in n-butanol before use. A few drops of 0.1 M aqueous solution of $AgNO_3$ (Daejung Chemicals & Metals Co.) were added to the solution of the product to observe halide ion precipitation, which was an intermediate species and an impurity in the final product. Nuclear magnetic resonance (NMR, Bruker Avance-250, 250 MHz) measurements were performed for the final identification of the product. The solvent for the NMR measurements was dimethyl sulfoxide-d_6 (DMSO-d_6, Cambridge Isotope Laboratories, Inc.).

All other ammonium salts were prepared in the same way from the following starting materials. The materials for N-ethyl-N, N-dimethyl-N-methoxymethylammonium tetrafluoroborate (Entry 4) were ethyldimethylamine (Tokyo Chemical Industry Co. Ltd, 98.0%) and chloromethylmethylether. N-ethyl-N,N-dimethyl-N-ethoxymethylammnonium tetrafluoroborate (Entry 6) was prepared from ethyldimethylamine and chloromethylethylether (Tokyo Chemical Industry Co., 96.0%). Trimethylamine and bromopropane (Tokyo Chemical Industry Co., 98.0%) were the starting materials for N,N,N-

trimethyl-N-propylammonium tetrafluoroborate (Entry 1). N-ethyl-N,N-dimethyl-N-propylammonium tetrafluoroborate (Entry 3) was prepared from ethyldimethylamine and bromopropane. N-ethyl-N,N-dimethyl-N-butylammonium tetrafluoroborate (Entry 5) was prepared from ethyldimethyl amine and 1-bromobutane(Tokyo Chemical Industry Co., 98.0%). The synthesized salts were dried overnight under vacuum before use.

The results for ^{1}H-NMR (250 MHz, DMSO-d_6) are as follows: Entry 2: δ 2.96(s, 9H), 3.60(s, 3H), 4.56(s, 2H); Entry 4: δ 1.21(t, 3H), 2.89(s, 6H), 3.28(q, 2H), 3.58(s, 3H), 4.56(s, 2H); Entry 6: δ 1.19(m, 6H), 2.89(s, 6H), 3.27(q, 2H), 3.79(q, 2H), 4.60(s, 2H); Entry 1: δ 0.88 (t, 3H), 1.67 (m, 2H), 3.01(s, 9H), 3.20 (q, 2H); Entry3: δ 0.88(t, 3H), 1.21(m, 3H), 1.64(m, 2H), 2.94(s, 6H), 3.15(m, 2H), 3.27(q, 2H). Entry 5: δ 0.19(t, 3H), 1.20(t, 3H), 1.27(m, 2H), 1.60(m, 2H), 2.94(s, 6H), 3.18(m, 2H), 3.29(q, 2H).

Cell preparation: The active material for the EDLC electrode was an activated carbon (MSP 20, Kansai Coke & Chemicals). The electrodes were made of an emulsion binder of styrene butyl rubber (SBR, Zeon, BM-400B) and carboxymethylcelluose (CMC, Sigma Aldrich) with Super-P black (MMM, Belgium) as the conducting agent. The active materials, super-P black, SBR, and CMC were mixed at a weight ratio of 81:12.8:4.2:2 to make a slurry. Deionized water was the solvent for the slurry. The slurry was spread on Al foil (23 μm thickness) and dried for 12 h at 80 °C under vacuum. The dried electrodes were roll-pressed to a thickness of 200 μm. The electrodes were punched out at a 14-mm diameter and then dried in a vacuum oven. All salts were dissolved in acetonitrile (AN, J. T. Baker. 99.8%) to prepare 1.0 M electrolyte solutions. Cell tests were performed using 2032-type coin cells (Hohsen Co.). Two carbon electrodes were used for the full cell tests. A 40-μ-thick cellulose separator was placed between the two electrodes. The cells were assembled in a dry box.

Instruments: Cyclic voltammetry (CV) was performed with the coin cells between 0 and 3.0 V using a potentiostat (model SP-150, Biological Science Instruments). A cell test cycler (PNE solution Co.) was used for the galvanostatic cell tests. Cell performances were evaluated using a galvanostatic charge and discharge procedure. Initially, cells were charged to 3.0 V by a constant current. Then, a constant potential of 3.0 V was maintained until the current was 1/10 of the charge current. Discharge started immediately after the constant potential charge mode was complete. The discharge current was the same as the charge current. The cut-off potentials were 0 and 3.0 V for the discharge and charge cycles, respectively. Cell capacitance was calculated from the following equation:

$$C = I / (\Delta V/\Delta t)$$

Using a galvanostatic charge/discharge process, the cell capacitance was calculated from the current, I, (in ampere (A)) and the slope of the discharge curve ($\Delta V/\Delta t$). The specific capacitances C_{am} in Farad per gram of electrode ($F \cdot g^{-1}$) for all samples were obtained from the following equation.

$$C_{am} = 2C / m_{am}$$

where m_{am} is the mass of active material (AC) in an electrode. In a symmetrical cell, the mass of each electrode is almost the same. The value of m_{am} was obtained from the average mass of activated carbon in the two electrodes in the cell. The slope from 0 to 3.0 V was used to calculate the discharge capacitance from galvanostatic tests. The influence of the IR drop drastically increases as discharge current increases. The capacitance values are averages of three measurements. Floating durability tests were carried out to observe the relative stability of cells under aggressive aging conditions. Durability test conditions for the cells we have were; holding 3.0 V for 24 h at 50°C.

Conductivity measurements were performed using a conductivity meter (Mettler Toledo, Seven Go Pro SG7-FKS) at various temperatures. (Concentration of solution: 1.0 M) A four-electrode conductivity probe (Mettler Toledo, Seven Go Pro Inlab 710) was calibrated with standards (Mettler Toledo) prior to the measurements. Samples were placed in a jar with a rubber seal on the screw cap. The probe was inserted through a tight rubber hole on the cap to prevent the entrance of moisture and the evaporation of the solvent under the measurement conditions. After each use, the probe was cleaned with nitric acid, rinsed with deionized water, and dried. The viscosities of the electrolytes were obtained with a capillary viscometer (SI Analytics GmbH, 527 10) and a visco-clock (SI Analytics GmbH). (Concentration of solution: 1.0 M) The viscosity value for each electrolyte was an average value of three measurements. The N_2 (99.999%) adsorption–desorption isotherms of the activated carbon were obtained by using a gas analyzer (MicrotracBEL Corp., BELSORP-max). Prior to analysis, the activated carbon was outgassed at 300 °C for 4 h under vacuum. The surface area was determined by the Brunauer–Emmett–Teller (BET) method. The total pore volume and the surface area were obtained from the region between 0 and 0.05 relative pressure. The surface area and pore size distribution for the activated carbon used in our work are provided in Figure S2 and Table S1. From the measurement, 90.4% of the pores were identified as micro pores.

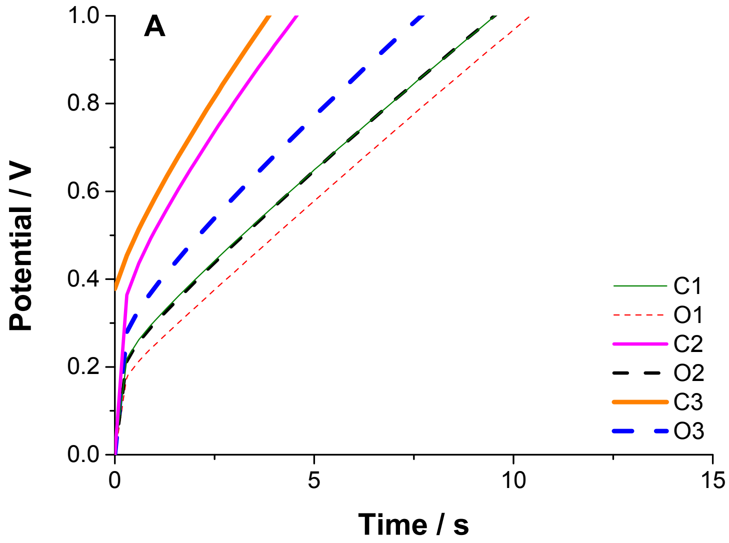

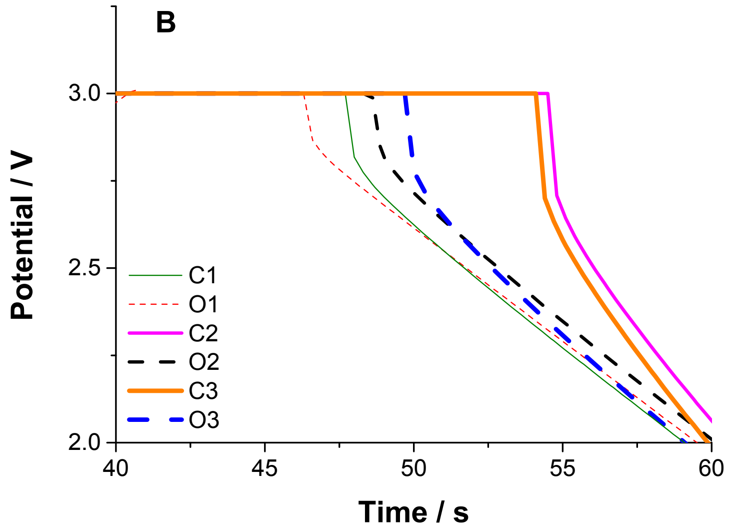

Figure S1. The enlargements of early stage of charge (A) and discharge (B) of Fig. 4A. IR drop values are obtained from these curve and enlisted in Table 1.

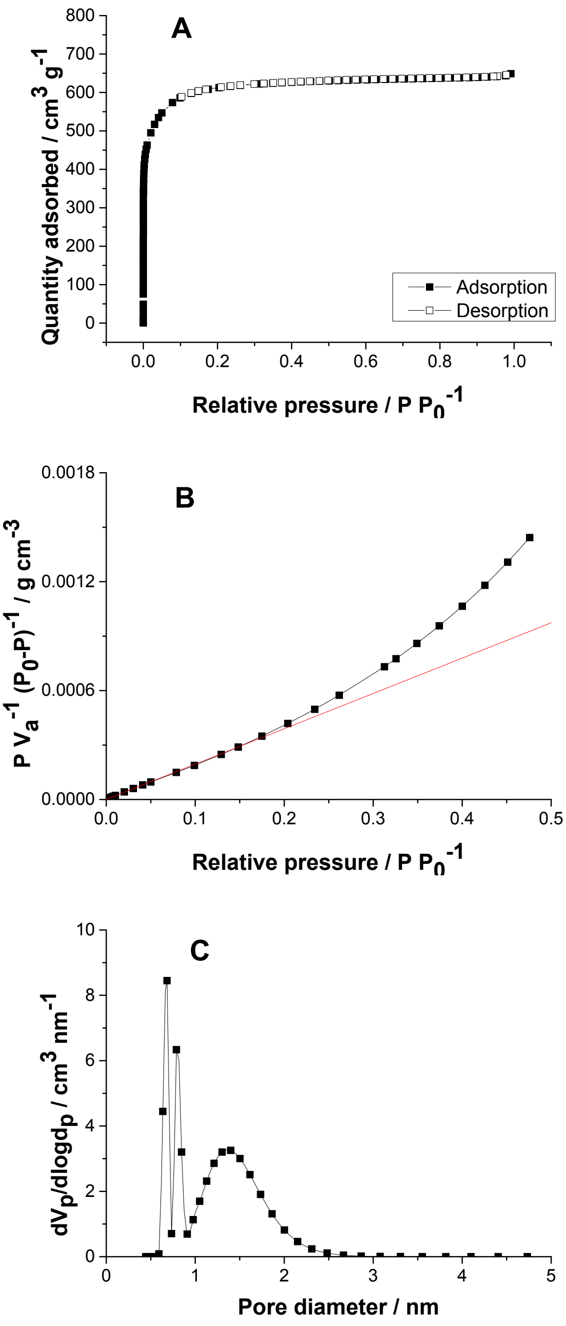

Figure S2. Analysis of pore structure with the activated carbon (MSP20).

(A) N_2 adsorption/desorption isotherm. (B) BET plot. (C) Pore size distributions obtained through the grand canonical monte carlo (GCMC) method.

Table S1. Pore properties of the activated carbon (MSP20).

Sample	S_{BET}[a]	V_T[b]	P_r [c]	
			micro	meso & macro
MSP20	2236.3	1.00	90.4	9.6

[a] Specific surface area (2236.3 $m^2 \cdot g^{-1}$) [b] Total pore volume ($cm^3 \cdot g^{-1}$) [c] Pore ratio (%)

Table S2. Capacitance and internal resistance changes of cells before and after durability test treatments for floating durability tests. The floating durability conditions are 3.0V, 24h and 50 °C. The current density is 0.1 A g^{-1} for the charge and discharge cycles.

Electrolytes	C_0[a] (F g-1)	C_d/C_0[b] (%)	R_0[c] (Ω)	ΔR[d] (Ω)
TEA BF_4	157.3	95.0	4.7	0.8
Entry 1	160.2	95.6	4.0	1.6
Entry 2	163.3	93.0	4.0	4.0

[a] initial capacitance (F g^{-1}), [b] capacitance retention (%), [c] initial internal resistance (Ω), [d] increase of internal resistance (Ω). The initial capacitance was obtained at 25°C. C_d is the capacitance after the durability treatments.
